# Nurse empowerment through Pharmacogenetics*

**DOI:** 10.1590/1518-8345.3415.3265

**Published:** 2020-08-12

**Authors:** Jordana Carvalhaes de Moraes, Fernanda Daniela Dornelas Nunes, Fernanda Borchers Coeli-Lacchini, Anderson Heiji Lima Miyazaki, Milena Flória-Santos, Riccardo Lacchini

**Affiliations:** 1 Hospital das Clínicas de Ribeirão Preto, Ribeirão Preto, SP, Brazil.; 2 Universidade de São Paulo, Escola de Enfermagem de Ribeirão Preto, Ribeirão Preto, SP, Brazil.; 3 Scholarship holder at Conselho Nacional de Desenvolvimento Científico e Tecnológico (CNPq), Brazil.; 4 Universidade de São Paulo, Faculdade de Ciências Farmacêuticas de Ribeirão Preto, Ribeirão Preto, SP, Brasil.; 5 Scholarship holder at Coordenação de Aperfeiçoamento de Pessoal de Nível Superior (CAPES), Brazil.; 6 GASEO Medicina Ocupacional, Ribeirão Preto, SP, Brazil.

**Keywords:** Cytochrome P-450 CYP2C9, Pharmacogenetics, Drug-Related Side Effects, Adverse Reactions, Nursing, Nursing Process, Citocromo P-450 CYP2C9, Farmacogenética, Efeitos Colaterais, Reações Adversas Relacionados a Medicamentos, Enfermagem, Processo de Enfermagem, Citocromo P-450 CYP2C9, Farmacogenética, Efectos Colaterales, Reacciones Adversas Relacionados con Medicamentos, Enfermería, Proceso de Enfermería

## Abstract

**Objective:**

to verify the existence of elements that justify the use of pharmacogenetics by the Brazilian nurse.

**Method:**

this is a quantitative, cross-sectional, observational, descriptive study, whose final sample was 67 individuals. The participants were healthy at the time of the study and reported a history of previous use and the occurrence of adverse effects by drugs commonly used and metabolized by CYP2C9. We collected 4 mL of venous blood for subsequent DNA extraction by salting out method and genotyping of the CYP2C9*2 and CYP2C9*3 polymorphisms, using Polymerase Chain Reaction in real time using Taqman assays.

**Results:**

the use of drugs metabolized by CYP2C9 was frequent (more than 75% of the individuals have already used between 2 or 4 of these drugs). Regarding adverse events, there were 19 perceived symptomatic occurrences associated with drugs metabolized by CYP2C9. The allele frequency of the polymorphism * 2 and * 3 in the population studied was 11.1% and 7.5%, respectively, and there was a coincidence between the presence of alleles of low enzyme activity and the occurrence of adverse effects.

**Conclusion:**

there are elements that justify the adoption of pharmacogenetics in the nursing care to reduce the occurrence of adverse reactions to drugs metabolized by CYP2C9.

## Abstract (pt)

**Objetivo:**

verificar a existência de elementos que justifiquem o uso da farmacogenética pelo enfermeiro brasileiro.

**Método:**

trata-se de um estudo quantitativo, do tipo transversal, observacional descritivo, cuja amostra final foi de 67 indivíduos. Os participantes estavam saudáveis no momento do estudo e reportaram histórico de uso prévio e ocorrência de efeitos adversos por fármacos comumente utilizados e metabolizados pela CYP2C9. Coletamos 4 mL de sangue venoso para posterior extração de DNA por método *salting out* e genotipagem dos polimorfismos CYP2C9*2 e CYP2C9*3 através de *Polymerase Chain Reaction* em tempo real, utilizando ensaios Taqman.

**Resultados:**

o uso de fármacos metabolizados pela CYP2C9 foi frequente (mais de 75% dos sujeitos já utilizaram entre 2 ou 4 desses fármacos). A respeito dos eventos adversos, houve 19 ocorrências sintomáticas percebidas, associadas a fármacos metabolizados pela CYP2C9. A frequência alélica do polimorfismo *2 e *3 na população estudada foi de 11,1% e 7,5%, respectivamente, com coincidência entre a presença dos alelos de baixa atividade enzimática e ocorrência de efeitos adversos.

**Conclusão:**

existem elementos que justificam a adoção da farmacogenética no cuidado do enfermeiro com objetivo de redução da ocorrência de reações adversas a fármacos metabolizados pela CYP2C9.

## Abstract (es)

**Objetivo:**

verificar la existencia de elementos que justifiquen el uso de la farmacogenética por parte del enfermero brasileño.

**Método:**

se trata de un estudio cuantitativo, transversal, observacional, descriptivo, cuya muestra final fue de 67 individuos. Los participantes estaban sanos en el momento del estudio e informaron un historial de uso previo y la aparición de efectos adversos por fármacos comúnmente utilizados y metabolizados por el CYP2C9. Recolectamos 4 ml de sangre venosa para la posterior extracción de ADN mediante el método de salazón y genotipificación de los polimorfismos CYP2C9 * 2 y CYP2C9 * 3 a través de la reacción en cadena de la polimerasa en tiempo real utilizando ensayos Taqman.

**Resultados:**

el uso de drogas metabolizadas por el CYP2C9 fue frecuente (más del 75% de las personas ya han usado entre 2 o 4 de estas drogas). Con respecto a los eventos adversos, hubo 19 casos sintomáticos percibidos asociados con medicamentos metabolizados por el CYP2C9. La frecuencia alélica del polimorfismo * 2 y * 3 en la población estudiada fue de 11.1% y 7.5%, respectivamente, y hubo una coincidencia entre la presencia de alelos de baja actividad enzimática y la aparición de efectos adversos.

**Conclusión:**

existen elementos que justifican la adopción de la farmacogenética en el cuidado del enfermero para reducir la aparición de reacciones adversas a los medicamentos metabolizados por el CYP2C9.

## Introduction

It is estimated that the so-called Adverse Drug Reactions (ADRs), which are between the 4^th^ and 6^th^ biggest causes of mortality in the USA, result in a percentage of hospital admissions around or above 10% in several countries and lead to a 15 to 20% of the hospital budget is spent to deal with complications resulting from medication use^(^ 1 ^)^. All ADRs are harmful, unintended response and the result of using a therapeutic dose of any medication. The consequences resulting from ineffective treatments or hospitalizations for drug poisoning are enormous and show the great importance of personalization and rationalization in the use of medications for both patients and health professionals^(^ 2 ^)^. The main causes for drug poisoning at therapeutic doses involve drug-drug interactions, drug-food interactions, patient health conditions (for example, liver or kidney failure) and the individual’s genetic characteristics^(^ 3 ^)^.

The metabolism of drugs in the human body occurs mainly through the hepatic cytochrome P450 (CYP) system^(^ 4 ^)^. These enzymes can have their activity and expression modulated by endogenous factors, such as genetic polymorphisms^(^ 2 ^-^ 3 ^,^ 5 ^)^, which are variations in the genetic code of individuals. These are hereditary characteristics, explaining family vulnerabilities to toxic reactions in the use of certain drugs^(^ 3 ^)^.

Of the liver enzymes of cytochrome P450, the CYP2C9 is one of the most important, responsible for the metabolization of drugs such as Aspirin and Diclofenac, Nonsteroidal Anti-inflammatory (NSAIDs), Losartan, Phenytoin, Tolbutamide, Warfarin, among others^(^ 6 ^)^. The gene encoding this enzyme (*CYP2C9*) has several important genetic polymorphisms, which can alter the metabolic profile of each individual, generating the following phenotypes: slow metabolizers (individuals with a decrease or absence of the metabolizing enzyme), intermediate (individuals with a slower than normal metabolism, but still present), extensive (individuals with regular metabolism – corresponds to the majority of the population), and ultra-fast (individuals with increased metabolizing enzyme production)^(^ 6 ^-^ 7 ^)^.

The importance of polymorphisms is clear when considering a drug with a narrow therapeutic window such as Warfarin. This anticoagulant can lead to hemorrhagic events in patients with alleles associated with reduced metabolism of the warfarin, for example, CYP2C9 * 2 and * 3^(^ 8 ^-^ 11 ^)^. In several countries, genetic testing prior to drug administration is already adopted to establish the correct dose of warfarin^(^ 9 ^-^ 10 ^)^, and efforts are being made to adapt this to the Brazilian reality^(^ 8 ^,^ 11 ^)^. This has been studied from a multiprofessional point of view, but it is important to understand how nurses can use this tool in their daily lives.

The nursing professional is responsible for performing many competencies, among which we highlight three central care activities: 1) medication administration, 2) the nurse’s management skills, important, among other things, to identify the cost-benefit of a health action, and 3) the education of the patient about the illness he/she has. These skills can be improved by incorporating pharmacogenetics in the nursing process^(^ 12 ^)^.

It is assumed that the Nursing Process is a method to systematize care and thus, in addition to allowing personalization of care, it unites nurses, patients, family and community. The Nursing Process is classified into five sequential and interrelated steps: data collection, diagnosis, planning, implementation and evaluation^(^ 13 ^)^. Studies have identified that the nursing process is capable of providing autonomy for the professional nurse, given that it enables the planning and organization of the actions of this team, enabling improvement and efficiency in the care provided, reduction of complications, length of stay in the hospital and the cost of care^(^ 14 ^)^. Pharmacogenetics can be incorporated in almost all stages of the nursing process. In the data collection stage, it allows the health team to detect the individual’s genetic data, outlining a diagnosis that will guide care planning^(^ 12 ^)^. Thus, in the same way that it is necessary to obtain local information to adapt concepts of pharmacogenetics to our population that has a mixed ethnic origin^(^ 8 ^,^ 11 ^)^, it is necessary to understand whether and how this knowledge can be applied to the nurse’s daily life.

In other countries, there is already evidence that this is important in this professional’s daily life, such as the use of genetic biomarkers in the care of diabetic^(^ 15 ^)^ and schizophrenic patients^(^ 16 ^)^, in the treatment of pain^(^ 3 ^)^, in the treatment of cancer^(^ 17 ^)^, but there is still no clear evidence that this is relevant in our context. Thus, the general objective of the present study was to verify the existence of elements that justify the use of pharmacogenetics by the Brazilian nurse. We evaluated three main pieces of information: 1) the frequency of use of drugs metabolized by CYP2C9 and the occurrence of adverse effects by the general population; 2) the frequency of CYP2C9 low activity alleles determined experimentally in our sample and its comparison with world data; 3) cost-benefit analysis to verify the feasibility of offering this information in the university context.

## Method

This is a quantitative, cross-sectional, observational and descriptive study, approved by the Research Ethics Committee (Portuguese acronym: CEP) of the Ribeirão Preto College of Nursing, University of São Paulo, under protocol number 55199116.1.0000.5393. All study participants signed the Free and Informed Consent Form (FICF). Associated with this research project, we developed educational material in an extension project^(^ 18 ^)^, and we have used this material to make the population aware of the use and importance of pharmacogenetics, with the intention that patients take the demand to health professionals and they start using pharmacogenetics on a routine basis.

Individuals of both genders, over the age of 40, attending the Ribeirão Preto campus of the University of São Paulo (USP) were eligible for the study. The option of approaching people over 40 is justified by the fact that it is demonstrated by the literature that with the advancing age there is a greater probability that the individual has already used pharmacotherapy at some point in life, or is currently using it. We did not use exclusion criteria in this study.

During the period of data collection, which occurred between the months of July and August 2016, the population invited to participate in the study was composed of 70 volunteers, three of whom did not accept the invitation. There was no exclusion or loss of any participant in the research, and therefore 67 people made up the study sample.

A questionnaire was applied to collect sociodemographic data to characterize the sample and, subsequently, an intravenous puncture was performed to collect 4 mL of venous blood, which was used for DNA extraction. The DNA was extracted by the salting out method, quantified by spectrophotometry, diluted and stored in a freezer at -20ºC. The determination of the genotypes referring to the polymorphisms * 2 and * 3 (rs1799853 and rs1057910, respectively) was made by Polymerase Chain Reaction (PCR) in real time using Taqman assays designed by the manufacturer (assays C__25625805_10 and C__27104892_10, respectively). The reactions were performed on the StepOne Plus equipment (Applied Biosystems, Carlsbad, CA, USA), and analyzed by the manufacturer’s software.

The cost-benefit analysis considered the unit costs of the materials used for the exam, normalized by the number of exams that each material allows to perform. The cost of labor or structural costs were not included in the expenses, as the objective of the analysis is to verify the feasibility of extension initiatives carried out in a university environment for the provision of genetic information to patients seen in its environment.

Regarding the statistical analysis, Hardy-Weinberg equilibrium deviation analyses and comparison of allelic frequencies between populations were performed using the Chi-Square test. The allele frequencies obtained in the study were compared with those reported from other populations by the HapMap project (https://www.ncbi.nlm.nih.gov/probe/docs/projhapmap/). Were selected populations of European (CEU) and African (YRU) origin, as they represent the main origins of the Brazilian population.

## Results

Regarding sociodemographic data (Table 1), the sample of this study was composed of Brazilians (100%), predominantly women (62.7%), aged between 40 and 49 years (43.3%), self-reported white (76.1%) and having completed high school (43.3%).

**Table 1:** Sociodemographic characteristics of the study participants (n = 67). Ribeirão Preto, SP, Brasil, 2016

Variables	n*	%^†^
Gender		
Female	42	62,7
Male	25	37,3
Age		
Average ± SD^‡^	52 ± 9	
Skin color		
White	51	76,1
Non-white	16	23,9
Nationality		
Brazilian	67	100

* n = Values in number of observations;

† % = Percentage frequency;

‡ SD = Standard deviation

Table 2 shows the reported data regarding the use of drugs metabolized by CYP2C9 enzyme. The most common types of drugs used by the participants were non-steroidal anti-inflammatory drugs. Therefore, the most common adverse effect was stomach pain, which is usual in this class. Our data indicate that the sample, although not subjected to chronic treatments, was exposed almost completely (98.5%) to drugs that were metabolized by *CYP2C9* (Table 2). In addition, a large number of patients (almost 29%) reported adverse reactions to medications, although they may be considered to be of low intensity. Interestingly, only five patients out of 25 classified as intermediate metabolizers had adverse reactions, which did not differ from extensive metabolizers (P = 0.270). Unfortunately, the number of individuals included did not allow us to stratify patients according to the different types of adverse drug reactions.

**Table 2:** Use of medications and occurrences of adverse events by study participants (n=67). Ribeirão Preto, SP, Brasil, 2016

Parameter	N (%)*
Previously used drugs metabolized by CYP2C9	66 (98.5%)
Ibuprofen	32 (17.2%)
Acetylsalicylic Acid	54 (29%)
Diclofenac	55 (29.6%)
Celecoxib	12 (6.5%)
Rosuvastatin	12 (6.5%)
Prasugrel	1 (0.5%)
Phenytoin	1 (0.5%)
Irbesartan	0 (0%)
Losartan	17 (9.1%)
Warfarin	2 (1.1%)
Number of medications already used	
= 1	10 (15.2%)
2 - 4	50 (75.8%)
>4	6 (9%)
Occurrence of adverse events	19 (27.5%)
Nausea	1 (5.3%)
Headache	1 (5.3%)
Vertigo	1 (5.3%)
Decreased urinary output	1 (5.3%)
Pain in the legs	1 (5.3%)
Paresthesia	2 (10.4%)
Stomach pain	8 (42.1%)
Allergic reactions	4 (21%)
Medications related to the occurrence of adverse events	
Aspirin	9 (42.8%)
Diclofenac	8 (38.1%)
Losartan	2 (9.5%)
Rosuvastatin	1 (4.8%)
Celecoxib	1 (4.8%)

* n (%) = Values in number of observations or percentage frequency

Table 3 shows the frequency of the genotypes obtained in the present study and Table 4 shows the allele frequency and the comparison with the frequencies of other populations of different ethnic origins (data from HapMap). In our sample, no individuals were found to have both * 2 and * 3 allele and homozygous * 2 or * 3, simultaneously. It is important to realize that of the 67 individuals studied, 25 (37%) have genotypes associated with the CYP2C9 intermediate metabolism phenotype.

**Table 3:** Genetic characteristics of the study participants (n = 67). Ribeirão Preto, SP, Brasil, 2016

Polymorphism	Genotype	Phenotype	n*	%^†^
*CYP2C9* * *2 (rs1799853)*	C/C (*1/*1)	EM^‡^	52	77.6
	C/T (*1/*2)	IM^§^	15	22.4
*CYP2C9* * *3 (rs1057910)*	A/A (*1/*1)	EM^‡^	57	85.1
	C/A (*1/*3)	IM^§^	10	14.9

* n = Values in number of observations;

† % = Percentage frequency;

‡ EM = Extensive metabolizer;

§ IM = Intermediate metabolizer (slower)

**Table 4:** Allele frequencies found in the studied population compared to allele frequencies of populations in the HapMap study (Caucasian and African origins). Ribeirão Preto, SP, Brasil, 2016

Polymorphism	Alele	n*	RP(%)^†^	CEU(%)^‡^	YRU(%)^§^	Pa^‖^	Pb^¶^
*CYP2C9* * *2 (rs1799853)*	*1	119	88.8	89.6	100	-	-
	*2	15	11.1	10.4	0	0.818	<0.001* *
*CYP2C9* * *3 (rs1057910)*	*1	124	92.5	94.2	100	-	-
	*3	10	7.5	5.8	0	0.774	0.007* *

* n = Values in number of observations;

† RP = Population of Ribeirão Preto, São Paulo;

‡ CEU = caucasian europeans - population of Caucasian origin composed of descendants of Europeans who inhabit the Utah region in the United States (USA);

§ YRU = *Yorubans* - population of African origin from the Yoruba ethnic group, inhabitants of Ibadan, Nigeria;

‖ Pa = p-value of the comparison between populations RP vs. CEU;

¶ Pb = p-value of the comparison between populations RP vs. YRU;

** Statistically significant

The allele frequency of the CYP2C9 * 2 and CYP2C9 * 3 polymorphism in the study population was 11.1% and 7.5%, respectively (Table 4). Our data indicate that although this population is mixed, the allele frequencies of CYP2C9 are much closer to those reported in European ancestry (P> 0.05) than those reported in populations of African ancestry (P <0.05).

Regarding the feasibility of genetic testing within a university extension program, Table 5 provides an estimate of costs. This was a very interesting scenario, since it was possible to take advantage of a pre-existing structure that is usually focused on research and post-graduate studies.

**Table 5:** Estimated price for genotyping in a situation of service provided within a public university. Ribeirão Preto, SP, Brasil, 2016

Item	Catalog number	Value paid*	Price per exam^†^
Taqman Probes	4362691	3,600.00	R$ 9.57
Master Mix	4371357	4,000.00	R$ 2.00
Collection Material	-	-	R$ 1.50
Value of DNA extraction^‡§^	-	-	R$ 8.00
Total			R$ 21.07

* Values obtained from www.thermofisher.com in 10/04/2019;

† The following items were included for this cost analysis: Taqman probes and respective Master Mix for genotyping; Latex gloves and vacutainer tubes; phenol and Eppendorf-type plastic tubes;

‡ DNA = Deoxyribonucleic acid;

§ Approximate values - have been rounded up since there are other low-cost items that are also used, in addition, personnel and equipment costs, not necessary in a context of service provided by the university, have been excluded from this calculation

## Discussion

The clinical implementation of pharmacogenetics is a challenge worldwide and has been very well accepted in cases where: 1) the evidence of the genetic association is reproducible and consistent, and 2) the clinical consequences of the presence of a certain allele are serious, for example, slow metabolizers using drugs with a narrow therapeutic window^(^ 8 ^,^ 11 ^)^. For the implementation, tests are required for each local population, and most of the scientific evidence is from Caucasian and North American populations. In fact, in North American society and on the European continent, the adoption of pharmacogenetics has been a reality for some years^(^ 19 ^-^ 20 ^)^, for example, with the recommendation of genotyping of the * 2 and * 3 alleles in patients before starting therapy with various drugs such as Warfarin, Abacavir and Tamoxifen. In addition, the area of oncology is intensively incorporating the study of the genetic bases of cancer and, consequently, specific pharmacological approaches for patients with certain risk alleles^(^ 17 ^)^. The challenge of implementing pharmacogenetics in clinical practice has several obstacles^(^ 8 ^,^ 11 ^)^, which have been faced in a multidisciplinary way. There are several notes in the literature about the need for nurses to actively participate in this process, both to contribute to the consolidation of the tool itself and to enable its application to their daily lives^(^ 3 ^,^ 15 ^-^ 17 ^,^ 21 ^-^ 22 ^)^.

The nurse, when appropriating pharmacogenetics as a tool for care, can enhance the benefits of this care in three moments: 1) administration of the drug; 2) cost-benefit analysis of the implementation of this tool in the management of the health system; 3) empower, by means of educational actions, the patient himself/herself, who, knowing to carry alleles of slow metabolism, may throughout his/her life alert health professionals who may attend him/her. The use of the nursing process in this context will help to systematize the information and list the scientific evidences that guide the adoption of pharmacogenetics in certain practices when there is sufficient evidence and not in others, when the scientific data are still insipient. This includes recognizing which patient should be tested, how the tests are done, what can be learned from them, how it applies to practice and, finally, what is the best way to explain the results to patients^(^ 23 ^-^ 24 ^)^.

In the present study, the allele frequencies of the CYP2C9 * 2 and * 3 polymorphisms that we found were similar to the frequencies found in the three main Brazilian studies with this theme^(^ 25 ^-^ 27 ^)^, which also found that the Caucasian population has a frequency of variant alleles 2 to 3 times higher than the black population. In addition, the present research showed that the allelic frequency in Brazilians of the CYP2C9 enzyme polymorphisms studied here is similar to that found in the European Caucasian population since the statistical tests did not show significant difference between these populations, although more than 20% of the population in this study declare themselves to be non-white.

The alleles studied here are primarily responsible for leading to a decrease in CYP2C9 activity, generating the phenotype of slow (homozygous) or intermediate (heterozygous) metabolizer. The CYP2C9 * 2 polymorphism (rs1799853) is located in exon 3 of the CYP2C9 gene and consists of the exchange of a cytosine for a thymine at a specific position in the DNA sequence, promoting the replacement of the amino acid arginine with a cysteine at position 144 of the protein. The CYP2C9 * 3 polymorphism (rs1057910), located in exon 7 of the same gene, consists of the exchange of an adenine for a cytosine, leading to the replacement of an isoleucine with a leucine in the protein chain. An *in vitro* study^(^ 29 ^)^ involving the culture of cells modified with the human CYP2C9 wild gene or variants, showed that the presence of these alleles leads to a 20 to 96% decrease in enzyme activity. In addition, there is an increasing number of evidences that point to a significant increase in the chance of adverse events occurring in individuals with * 2 or * 3 alleles, such as hemorrhagic events with the use of warfarin^(^ 30 ^)^, confirming the functionality and relevant clinical implication of the * 2 and * 3 alleles. From our results, we can classify the heterozygous individuals for the genotyping of the * 2 and * 3 alleles (15 and 10, respectively) as having a high probability of being intermediate metabolizers for the CYP2C9 enzyme, that is, with a slower metabolism than the normal, although still present. Meanwhile, the other individuals, homozygous C/C and homozygous A/A, (carriers of the * 1 allele), can be considered as more likely to be extensive metabolizers of this enzyme. We cannot categorically define phenotypes since we have not exhausted the genetic characterization including other polymorphisms. Thus, the intermediate metabolizer classified only by * 2 and * 3 can actually be a slow metabolizer because it may have a variant in another polymorphism. Since more than 5-12%^(^ 31 ^-^ 32 ^)^ of the slow metabolizing phenotypes are explained only by the * 2 and * 3 alleles, we consider that the genetic classification made here has a high probability of corresponding to the phenotype.

It is interesting to note that 26% of the ADRs reported here occurred in patients with slow CYP2C9 alleles. This demonstrates that complex phenotypes such as pharmacogenetics are affected in a multifactorial way, that is, the genotype has no determinant relation to the phenotype (as in the case of monogenic diseases, for example)^(^ 33 ^-^ 34 ^)^. Several factors, such as treatment time, concomitant use of other substrates for the same enzyme, dose, age and health status, among other factors, are also associated with ADRs^(^ 35 ^)^. This explains why part of the patients with slow alleles did not have ADRs even when exposed to drugs that are substrates of CYP2C9, as well as the appearance of ADRs in non-carriers of the * 2 and * 3 alleles of CYP2C9.

The results reported here showed that stomach pain was the most common adverse event presented by the study participants, and that Aspirin was the drug most associated with the occurrence of adverse effects, which is in agreement with the literature^(^ 36 ^)^ that points out that the most important side effects of NSAIDs occur in the gastrointestinal tract; this explains the percentage of 20% of patients who cannot tolerate treatment due to these effects (such as stomach pain, for example). It is important to note that many patients do not experience pain, which further increases the risk of developing serious complications, such as bleeding and perforation of the stomach.

In addition, two of the ADRs reported by intermediate metabolizers were associated with the use of Aspirin and reported as allergic reactions, more specifically intense urticaria. This is similar to that found by other authors^(^ 37 ^)^ who, when analyzing the genotypes of 148 patients with urticaria associated with the use of Aspirin and 260 control subjects, found a significantly higher allele frequency of the * 2 and * 3 alleles in the group that reported urticaria intolerant to Aspirin.

The second drug most commonly used by the population in this study, and the second most related to the occurrence of adverse events, was Diclofenac. Although studies indicate that the use of this drug is common and generally well tolerated in the clinic, a research^(^ 38 ^)^ has already reported that the presence of genetic variants of the CYP2C9 enzyme, more specifically the CYP2C9 * 2 and CYP2C9 * 3 allele, considerably increases the risk of gastrointestinal disorders, including bleeding, during the use of NSAID other than Aspirin. In our study, two individuals, heterozygous for the * 2 allele, reported adverse reactions related to the use of Diclofenac, an NSAID, being one with allergic reaction and one with stomach pain.

In our results, an individual, heterozygous for the * 3 allele, reported an adverse reaction to the use of the drug Losartan, characterized as an allergic reaction (cough and throat irritation). A similar association has been found previously^(^ 39 ^)^, relating the presence of the * 3 allele as the most significant variant to predict enzyme activity, leading to a significant decrease in Losartan metabolism, while the * 2 allele seems to have less importance in determining the activity enzyme of CYP2C9. Other researchers^(^ 40 ^)^, studying 59 Caucasian individuals with chronic kidney disease and chronic use of Losartan, found allelic frequencies of the * 2 and * 3 variants of 5% and 6%, respectively, whose presence was directly related to an increase in proteinuria and in the non-reduction of systolic and diastolic blood pressure, confirming the need for studies on the role of the enzyme CYP2C9 and its variants not only in preventing adverse events from antihypertensive therapy with Losartan, as well as its effectiveness in clinical practice. In the sample of this study, 9.1% said they use current or have already used Losartan, while two individuals reported the occurrence of adverse effects related to this medication, confirming the importance of investigations relating the enzyme CYP2C9 to the metabolism of this antihypertensive.

Thus, our results show that with previous genotyping, five individuals could have benefited from the pharmacogenetic results. If the dosages were adjusted according to the genetic information, the adverse effects could probably be avoided since these individuals are intermediate metabolizers of CYP2C9. It is worth mentioning, however, that the future benefit of genotyping for the participants in this study may be even greater, since the remaining 20 intermediate metabolizing individuals may have ADR in the future when using drugs metabolized by CYP2C9, even though they have not reported them until the moment.

Currently, we are witnessing an increase in the number of medicines available in the Brazilian market and among the reasons that seek to explain this trend are the aging population, the increase in chronic non-communicable diseases and the popular belief that the only possibility of being healthy is to consume health, implying a high desire to consume medicines, one of the most important symbols of health in this society^(^ 2 ^,^ 41 ^)^. The results found in this study help to confirm this trend, since it was almost unanimous (98.5%) the previous or current use of medications metabolized by the enzyme CYP2C9.

In Brazil, it is estimated that 23% of the population consumes 60% of the national production of medicines, especially people over 60, whose other common characteristic is polypharmacy, that is, the use of four or more medicines^(^ 42 ^)^. In our study, polypharmacy was verified in more than 75% of the research participants.

Therapeutic failures and the occurrence of adverse events related to drugs contribute to the strong frequency and prevalence of hospital admissions. In this regard, a study conducted in three tertiary teaching hospitals in Japan found that approximately two thirds of adverse events and medication errors occurred in the ordering/separation phase, followed by monitoring (18.7%), administration (14% ) and dispensation (2.33%), greatly increasing the length of hospital stay^(^ 43 ^)^.

In this way, our study brings clear evidence that the genotyping of the * 2 and * 3 alleles is relevant in our population, and supports the application of the concepts of pharmacogenetics for the planning and administration of drugs, the dose of which can be evaluated in the context of “extensive metabolizers”, “intermediate metabolizers” and “slow metabolizers” to have a more accurate assessment of the risk that the patient is exposed in relation to ADRs.

We ratify that the administration of drugs is a fundamental care of the nursing professional and that it requires constant innovation within the nursing process. A domain of knowledge is necessary, which is complex because it involves drug interactions, mechanism of action and clinical complications arising from adverse effects. This is the competence of the nurse since he/she is the professional responsible for knowing the patient’s clinical history and needs to check the medical prescription before administering the medication^(^ 44 ^)^. Pharmacogenetics comes to add and subsidize nursing and patient empowerment^(^ 45 ^)^.

From the point of view of public health management, nurses have a crucial role in assessing the cost-benefit of applying this approach in professional practice. In fact, the urgent need to conduct this type of research in Brazil is clear^(^ 8 ^,^ 11 ^)^. In the present study, we tried to envision the role that the public university can play in promoting the beginning of the application of pharmacogenomics in the single health system by offering these tests at cost. In this context, we estimate the cost per patient of genotyping the * 2 and * 3 alleles at R$ 21.07, a value much lower than the average cost of a hospital stay in a medical clinic in the state of São Paulo in 2019, which was of R$ 1,433.16^(^ 46 ^)^.

If we consider that out of 67 participants, five had avoidable ADR with this genetic test, we could propose the following analysis: 67 patients multiplied by the cost of exams versus the cost of five potential hospitalizations. We can see that carrying out the genetic test costs less for SUS (Brazil’s Unified Health System) (R$ 1,411.69), than the cost of five hospitalizations (R$ 7,165.80). If we also consider that the genetic information does not change over the life of the patient, we can imagine that each test can, over time, avoid more than once severe ADRs, which further maximizes the economic benefit of adopting pharmacogenetics. Thus, the present study confirms the importance of partnerships between the academic field and the university hospital, for example, in view of the financial viability of investment in prevention versus care, already established in the literature^(^ 47 ^)^, and which can also be carried out by pharmacogenetic studies.

Corroborating this idea, a research carried out in Germany used the patient registry to calculate the financial costs, for 1 year, to treat patients diagnosed with schizophrenia, with or without pharmacogenetic analysis for polymorphisms in the CYP2D6 genes. The study concluded that prior genotyping was responsible for reducing 28% of spending on slow and ultrafast metabolizers. This evidence confirmed that these types of metabolizers generate higher costs to the health system than those that have the ancestral genotype for CYP2D6, and that these costs could be significantly reduced by pharmacogenetic tests prior to the start of therapy with antipsychotics and antidepressants^(^ 48 ^)^.

The importance of pharmacogenetic tests is more evident when viewing the amount of financial resources spent due to the increase in hospital stay caused by the occurrence of errors or adverse events. Thus, it is essential to highlight the results of a study carried out in the USA, which point out that the adjusted cost of hospitalization for patients who suffered some type of adverse event related to medications was US$ 1,851.43 higher than when compared to hospitalization without these events occurring^(^ 49 ^)^. The estimated total cost of hospitalization related to adverse events from 2008 to 2011 in the USA was US$ 142 billion. In 2011 alone, the total cost of hospitalization related to ADRs was estimated at US$ 38.9 billion^(^ 49 ^)^.

In addition to the financial value, we know how long hospitalizations generally are, including those caused by ADR, and represent a strain and suffering in the lives of patients and their families, which contributes to the worsening of quality of life and the affirmation of a hospital-centered and specialized health model, in opposition to the current trend of a system rebuilt through the axis of primary care and family health^(^ 47 ^)^.

The third axis of application of the data presented in this work concerns the educational role that nurses play when passing on knowledge to the patient and other professionals of the multidisciplinary health team.

Advocating for the patient is one of the most important care and one of the most well-known functions of the nursing professional^(^ 50 ^)^. The nurse has the opportunity to appropriate pharmacogenomic knowledge, educate the patient (providing the possibility of empowering him/her), and assist the multidisciplinary team in personalizing the therapy in such a way as to increase the effectiveness and safety of the pharmacological treatment^(^ 51 ^)^.

A key to health promotion since the Ottawa Charter in 1986, empowerment, can be understood as a process of training individuals and communities to take greater control over individual, socioeconomic and environmental factors that affect health^(^ 52 ^)^. Among the individual factors, we can infer that there are genetic information and the right to obtain and use that information. Given the real possibility and the powerful implications of the growth in the use of genetic information, it is necessary to educate the population in a general way, which, in addition to lacking basic notions of genetics and pharmacology, still needs to pay attention to ethical, family, social, legal and financial aspects that this information affects^(^ 53 ^)^.

It is important to highlight that the nursing process is characterized as a powerful opportunity to strengthen educational competence, essential in the nurse’s work process^(^ 54 ^-^ 55 ^)^. The nursing process can contribute in this sense, to the extent that it favors direct educational actions with patients and family members regarding adequate treatment, as well as important associated signs and symptoms.

A study developed in institutions in the state of Espírito Santo identified that actions taken with patients in a superficial way can disqualify the other stages of the nursing process^(^ 56 ^)^. On the other hand, a research carried out in Hungary has shown that empowered patients, identified as literate and trained, reported better health status compared to any other group of patients^(^ 57 ^)^. Empowerment also takes place with respect for nurses in all their levels of performance, since the domain of certain knowledge drives them towards a more effective and efficient health decision-making^(^ 21 ^,^ 58 ^-^ 59 ^)^. In fact, there is evidence that the use of pharmacogenetics in countries where this is implemented positively impacts the care process for both the nurse and the patient^(^ 60 ^)^.

Through teaching and pharmacogenetic empowerment, we will also be contributing to the formation of more sensitive health professionals, capable of perceiving each individual as unique and, in this way, adapting their form of assistance for each person, transforming the care relationship to the extent that the individual becomes an active subject of his/her treatment. It is also important to increasingly adhere to the nursing process, so that patients and family members can be educated so that they are protagonists of their own care process.

As a limitation of the study, we highlight that the relatively small sample does not allow the detection of rare alleles, but this was not a problem, since the * 2 and * 3 alleles are relatively common and explain most cases of reduced CYP2C9 metabolism. Another limitation refers to the fact that participants from the general population and not patients served by a health service were evaluated. On the one hand, this reduced the chance of identifying serious adverse effects, but it has the advantage of allowing the results to be visualized in the population more globally. Finally, the cost analysis was done in a specific context such as the university providing a service to society. Cost analyses involving the private sector are necessary, including salaries, training, acquisition of equipment and fixed costs such as electricity, water, physical space, among others.

## Conclusion

There are elements that point not only to the relevance, but also to the feasibility of applying pharmacogenetics in the context of Brazilian nursing. There is evidence of favorable cost-benefit and potential to benefit patients who could avoid adverse reactions to drugs.
